# Prevalence of dysphagia in multiple sclerosis and its related factors: Systematic review and meta-analysis

**Published:** 2018-10-07

**Authors:** Alireza Aghaz, Alireza Alidad, Ehsan Hemmati, Hussein Jadidi, Leila Ghelichi

**Affiliations:** 1 Department of Speech Therapy, School of Rehabilitation Sciences, Isfahan University of Medical Sciences, Isfahan, Iran; 2 Department of Speech and Language Pathology, School of Rehabilitation Sciences, Iran University of Medical Sciences, Tehran, Iran; 3 Student Research Committee, School of Advanced Technologies in Medicine, Iran University of Medical Sciences, Tehran, Iran; 4 Student Research Committee, Department of Biostatistics and Epidemiology, School of Health, Isfahan University of Medical Sciences, Isfahan, Iran; 5 Rehabilitation Research Center, Iran University of Medical Sciences, Tehran, Iran

**Keywords:** Dysphagia, Multiple Sclerosis, Prevalence, Systematic Review

## Abstract

**Background:** Dysphagia is the most prevalent sign of multiple sclerosis (MS) which can reduce the quality of life and augment mortality in the final stages of MS. We presented a systematic review to estimate the prevalence of dysphagia in general and separately for each evaluation method (subjective and objective), and to analyze the causes of this rampant disease.

**Methods:** Cross-sectional and prospective cohort studies were reviewed and scientific proofs were evaluated consistent with the pre-specified levels of certainty.

**Results:** Twenty-two articles entered the meta-analysis phase; the estimation of the general prevalence of dysphagia in MS-affected patients was 43.33% related to all the 22 studies. Moreover, the estimate of the prevalence via the subjective (16 studies) and objective (6 studies) methods were 37.21% and 58.47%, respectively.

**Conclusion:** This study obtained the prevalence rate of dysphagia in patients affected by MS globally, yet there was infinite statistical society and limited methodological quality. Thus, more extensive studies are required for a better understanding of the global epidemiology regarding dysphagia in MS.

## Introduction

Multiple sclerosis (MS) is an inflammatory disease of the central nervous system (CNS), destroying the myelin shield of the neurons.^1^ This progressive disease can occur at any age, while its primary emergence falls mainly between 20 and 40 years of age.^2^ MS in women is two to three times more prevalent than in men and is related to age, gender, and genetic, geographical, and racial factors.^3^ Depending on the extent, diversity of anatomic area, rate, and the beginning time of MS lesions, its clinical manifestations in such patients are different. Generally, disorders observed in this disease pertain to sensory systems, motion, vision, intestinal and bladder functions, and anomalies in cognition and physical capability.^1^

Dysphagia is the most prevalent sign in MS,^4^ the cause of which can be a combination of damages in certain structures such as corticobulbar nerve tract, cerebellum, brainstem, and lower cranial nerves^4^ and cognitive disorders.^5^ Dysphagia can reduce the quality of life and increase the risk of dehydration, which in turns leads to increased mortality in the last stages of the disease.^6^^,^^7^ Presently, the diagnostic methods of this disorder are divided into objective and subjective categories, each with its own tools, devices, and questionnaires. Accordingly, there are quite different and inhomogeneous estimations for the prevalence of dysphagia in individuals affected by MS. The aim of this study was to estimate the prevalence of dysphagia in general (without considering the evaluation method) and separately for each evaluation method, and to analyze the effective causes for the prevalence of this disorder with regards to the methodology of the reviewed studies.

## Materials and Methods

By assessing the titles and abstracts of the identified articles and after eliminating the repeated papers, the contents of the related articles were studied. It is to note that only original studies and systematic reviews were selected, and case studies, brief reports, and posters were not employed in this investigation.

**Figure 1:**
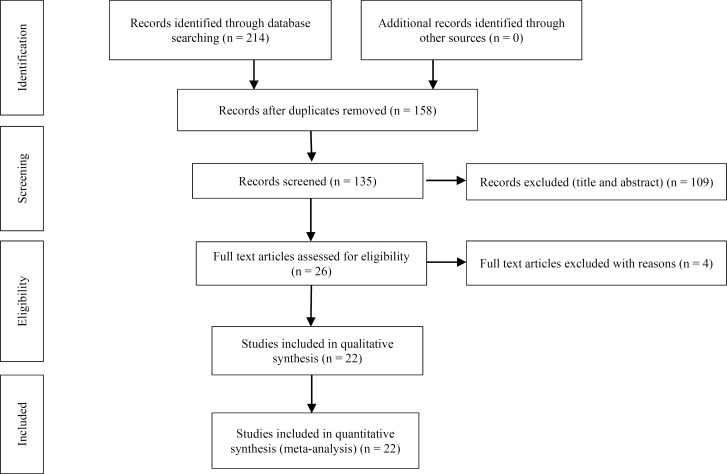
The flowchart of study selection process

We reported this systematic review and meta-analysis according to Preferred Reporting Items for Systematic Reviews and Meta-Analyses (PRISMA) statement^8^ and searched the texts with keywords like “prevalence”, “deglutition”, “dysphagia”, and “multiple sclerosis” in national and international scientific search engines (PubMed, Science Direct, Web of Science, and Google Scholar as well as Magiran, IranMedex, and SID) from 1980 to April 2018. The studies had the inclusion criteria suggested by the two authors who reviewed this study. Separately and with no prior information to each other’s investigations, the two authors analyzed the titles and abstract of different studies with regards to inclusion and exclusion criteria. Provided that the inclusion criteria for an article had not been approved with respect to the titles and abstracts by the authors, they would have analyzed the whole study. The final acceptance of the studies was based on the agreement of the two authors. In case of disputes in the analyses of the two authors, a third reviewer would evaluate the conflicts in conclusions, without being informed about the previous judgments. The references of the articles with inclusion condition and also the related articles were used to extract the required studies. Figure 1 illustrates the flowchart of the search and identification of the articles. 

The inclusion criteria were: 1. use of “Multiple Sclerosis” in the title of the articles, 2. use of quantitative values for the prevalence of dysphagia in the context of articles, 3. adult samples in the considered population, 4. articles that were not case reports, and 5. availability of the article for reviewing in English or Farsi. The studies that were merely in the form of posters and abstracts were excluded from the study.

Two authors separately extracted the required information from all the studies and summarized them in a table. We extracted the reported quantitative values related to the prevalence of dysphagia and other required information from the contents of the articles; these values were further standardized via a statistical specialist (when required) to provide the possibility for required evaluations and comparisons associated with the prevalence of dysphagia in MS.

The two authors separately assessed the quality of the related studies and agreed on the ones to be used. The disputes were solved by a third party. The features of the studies were the name of the first researcher, year of publication, geographical location, and the average age of the patients, all of which were identified and collected. Clinical variables extracted from the studies included the sample size, dysphagia diagnostic criteria (objective and subjective), the number of people affected by dysphagia, and disease disability based on the Expanded Disability Status Scale (EDSS), subtype, and duration. 

The standard error (SE) of each study was calculated with regards to the binomial distribution and the studies were combined considering the variance and the sample size. The confidence interval (CI) of 95% was used to calculate the point of prevalence and weigh each study. Due to inhomogeneity, the random effects model was employed so as to combine the studies. Moreover, Cochran test and 12 indexes were applied for evaluation of the inhomogeneity, and Stata software (version 12, Stata Corporation, College Station, TX, USA) was used for data analysis.

## Results

***Study selection*** *:* After browsing credible national and international websites, 214 papers were collected, out of which 79 repetitive articles were eliminated and 135 studies were included in the systematic stage. Following the analysis of the titles and abstracts of these studies, 109 papers were omitted and after investigating the contexts of the 26 remaining papers, 4 were also eliminated due to not meeting the inclusion criteria. Finally, 22 articles entered the meta-analysis phase (Figure 1).

***Study features: ***The considered studies were published in English language from 1981 to 2017. We categorized the 22 studies (with 5495 people) into “objective” and “subjective” groups according to the meta-analysis method. 16 studies were considered subjectively, and the 6 others were analyzed objectively. Moreover, 3 out of the 22 selected studies were “prospective cohort”, while 19 studies were “cross-sectional”. The mean age was in the range of 34-55 years, the sample size was between 18 and 1875, the mean disease duration of the studies ranged from 3.5 to 17 years, and the mean disability scored by EDSS varied from 1.8 to 7.4 (Tables 1 and 2).

**Figure 2:**
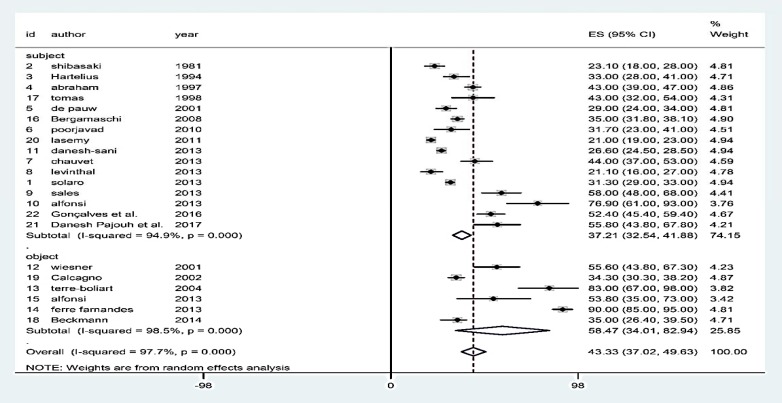
Rate of dysphagia prevalence in multiple sclerosis (MS)-affected patients and its 95% confidence interval (CI) in the considered studies based on the random effects model; the midpoint of each line shows the estimation of the prevalence and the length of the line indicates the 95% CI of each study. The rhombic sign shows the rate for the prevalence combination in the studies.

***Rate of prevalence*** *:* To calculate the general prevalence of dysphagia in patients with MS, the results of meta-analysis were obtained through the use of the random effects model. The prevalence estimates of dysphagia in the subjective and objective methods were 37.21% (95% CI: 32.54-41.88) and 58.47% (95% CI: 34.01-82.94), respectively. In all the studies, the estimation of the general prevalence of dysphagia in MS-affected patients was 43.33% (95% CI: 37.02-49.63). A considerable inhomogeneity was observed between the subjective group (P < 0.0001, I^2 ^= 93.2%) and the objective group (P < 0.0001, I^2 ^= 99.1%) (Figure 2).

***Correlations*** *: *Through the use of Spearman correlation coefficient, we further studied the correlation between the prevalence of dysphagia and three other variables, namely EDSS-based disease severity, duration of disease, and MS stages which were respectively reported in 14, 10, and 10 studies. The results of table 3 showed that there existed no significant relationship between the prevalence of dysphagia and the three variables.

**Figure 3:**
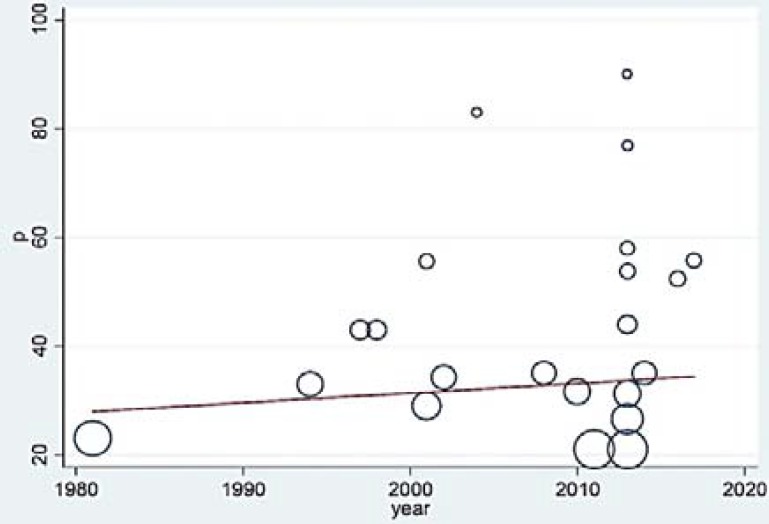
Meta-regression graph of dysphagia prevalence in the patients affected to multiple sclerosis (MS) according to the considered duration of the studies; the circles show the weight of the studies.

***Meta-regression*** *:* Meta-regression analysis was used to investigate the prevalence of dysphagia with regards to the duration of studies, sample size, and average age of the patients. According to the meta-regression graph, the rate of dysphagia in patients with MS augmented by increasing the duration of the studies (Figure 3).

**Table 1:** Prevalence of dysphagia in multiple sclerosis (MS) via subjective screening method

**References**	**Country**	**Study ** **design**	**Sample ** **size**	**Mean age ** **(year)**	**Diagnostic method**	**Disease ** **subtype (%)**	**Mean disease ** **duration (year)**	**Mean disease ** **disability (EDSS)**	**Prevalence ** **(%)**
Shibasaki, et al.^9^	UK	Cross- sectional	264	40.5	Coding sheet contained history and physical findings	-	10.3	-	23.1
Hartelius and Svensson^10^	Sweden	Cross- sectional	203	< 55.0	22 questionnaire tools	-	> 10 (70%)	-	33.0
Abraham, et al.^11^	USA	Cross- sectional	525	45.0	One-page dysphagia- screening questionnaire	-	-	5.2	43.0
Thomas and Wiles ^12^	UK	Cross- sectional	79	44.0	A 26-part questionnaire, drinking 150 ml water	-	11.8	6.0	43.0
De pauw, et al.^13^	Belgium	Cross- sectional	308	50.0	Johns Hopkins swallowing center	RRMS: (32), PPMS: (22), SPMS: (46)	17.0	6.5	29.0
Bergamaschi, et al.^14^	Italy	Cross- sectional	226	40.5	DYMUS questionnaire	-	10.1	3.1	35.0
Poorjavad, et al.^6^	Iran	Cross- sectional	101	34.0	NDPCS	RRMS: (74.3), PPMS: (6.9), SPMS: (18.8)	5.9	2.2	31.7
Lasemi, et al.^15^	Iran	Cross- sectional	400	34.2	EDSS	PPMS: (30.8), SPMS: (34.6)	< 7 (61.5%) > 7 (38.5%)	-	21.0
Levinthal, et al.^16^	USA	Cross- sectional	218	47.6	MDADI questionnaire	RRMS: (70.6), PPMS: (4.1), SPMS: (11.0)	13.3	-	21.1
Solaro, et al.^4^	Italy	Cross- sectional	1875	43.3	DYMUS questionnaire	RRMS: (69.0), PPMS: (7.0), SPMS: (24.0)	11.4	3.3	31.3
Sales, et al.^7^	Brazil	Cross- sectional	100	45.5	DYMUS questionnaire	RRMS: (66.0), PPMS: (18.0), SPMS: (16.0)	8.0	3.0	58.0
Alfonsi, et al.^17^	Italy	Prospective cohort	26	44.2	DYMUS questionnaire	RRMS: (34.6), PPMS: (30.8), SPMS: (34.6)	-	4.7	76.9
Danesh-Sani, et al.^18^	Iran	Cross- sectional	500	44.6	A standard neurological examination, EDSS	-	0-7 (63.2%) > 7 (36.8%)	-	26.6
Chauvet, et al.^19^	France	Cross- sectional	150	-	DYMUS questionnaire	-	-	-	44.0
Goncalves, et al.^20^	Brazil	Cross- sectional	34	38.0	NOT-S	-	-	-	55.8
Pajouh, et al.^21^	Iran	Cross- sectional	105	33.8	DYMUS questionnaire	-	3.5 ± 3.1 (mean ± SD)	1.8 ± 1.3 (mean ± SD)	52.4

RRMS: Relapsing remitting multiple sclerosis; PPMS: Primary progressive multiple sclerosis; SPMS: Secondary progressive multiple sclerosis; EDSS: Expanded disability status scale; NDPCS: Northwestern dysphagia patient check sheet; MDADI: MD Anderson dysphagia inventory; DYMUS: Dysphagia in multiple sclerosis; NOT-S: Nordic orofacial test-screening; SD: Standard deviation

**Table 2:** Prevalence of dysphagia in multiple sclerosis (MS) via objective screening method

**References**	**Country**	**Study design**	**Sample ** **size**	**Mean age ** **(year)**	**Diagnostic method**	**Disease ** **subtype (%)**	**Mean disease ** **duration (year)**	**Mean disease ** **disability (EDSS)**	**Prevalence (%)**
Wiesner, et al.^22^	Switzerland	Cross-sectional	18	47.0	Clinical and VFSS	-	-	6.2	55.6
Calcagno, et al.^23^	Italy	Cross-sectional	143	49.9	Direct examination and FEES	-	17.03	6.8	34.3
Terre-Boliart, et al.^24^	Spanish	Cross-sectional	23	-	Clinical and VFSS	-	-	7.4	83.0
Fernandes, et al.^25^	Brazil	Cross-sectional	120	38.5	Clinical and VFSS	RRMS: (65.8), PPMS: (5.0), SPMS: (29.2)	-	5.0	90.0
Alfonsi, et al.^17^	Italy	Prospective cohort	26	42.2	FEES	RRMS: (34.6), PPMS: (30.8), SPMS: (34.6)	-	4.7	53.8
Beckmann, et al.^26^	Turkey	Prospective cohort	51	32.2	EMG	RRMS: (100), PPMS: (0), SPMS: (0)	4.52	-	35.0

VFSS: Videofluoroscopic swallowing study; FEES*:* Fiberoptic endoscopic evaluation of swallowing; EMG: Electromyography; RRMS: Relapsing remitting multiple sclerosis; PPMS: Primary progressive multiple sclerosis; SPMS: Secondary progressive multiple sclerosis; EDSS: Expanded disability status scale

**Table 3:** Correlation between the prevalence of dysphagia and the severity of the disease, the duration of the disease, and the stages of multiple sclerosis (MS)

**Spearman**		**Mean disease ** **disability (EDSS)**	**Mean disease ** **duration (year)**	**Disease subtype**		
				**RRMS** ^*^	**PPMS** *	**SPMS** *
Prevalence of dysphagia	Correlation coefficient	0.11	-0.52	-0.26	0.26	0.18
	P*	0.7200	0.1800	0.5200	0.5200	0.6300
	N	14	10	10	10	10

EDSS: Expanded disability status scale; RRMS: Relapsing remitting multiple sclerosis; PPMS: Primary progressive multiple sclerosis; SPMS: Secondary progressive multiple sclerosis

* The significance level in this study is 0.5000.

**Figure 4:**
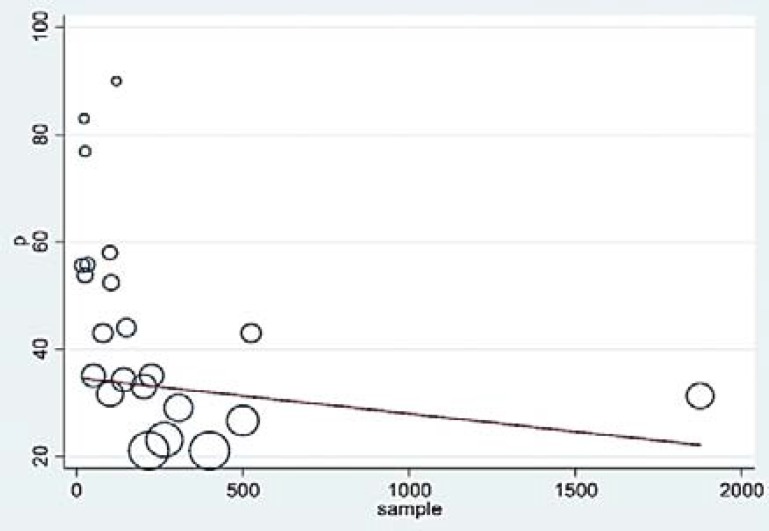
Meta-regression graph of dysphagia prevalence in the patients affected to multiple sclerosis (MS) according to the sample size; the circles show the weight of the studies.

However, the difference was not statistically significant (P = 0.1600). Moreover, with the increase in the sample size, the prevalence decreased (Figure 4), yet this difference was not significant either (P = 0.1600). 

**Figure 5:**
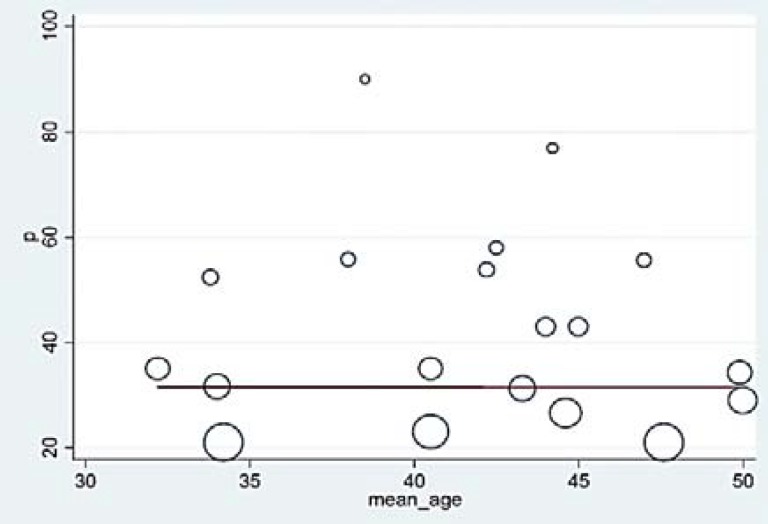
Meta-regression graph of dysphagia prevalence in the patients affected to multiple sclerosis (MS) according to the average age of the patients; the circles show the weight of the studies.

The average age of the patients was approximately constant according to the meta-analysis graph (Figure 5) (P = 0.9500).

**Figure 6:**
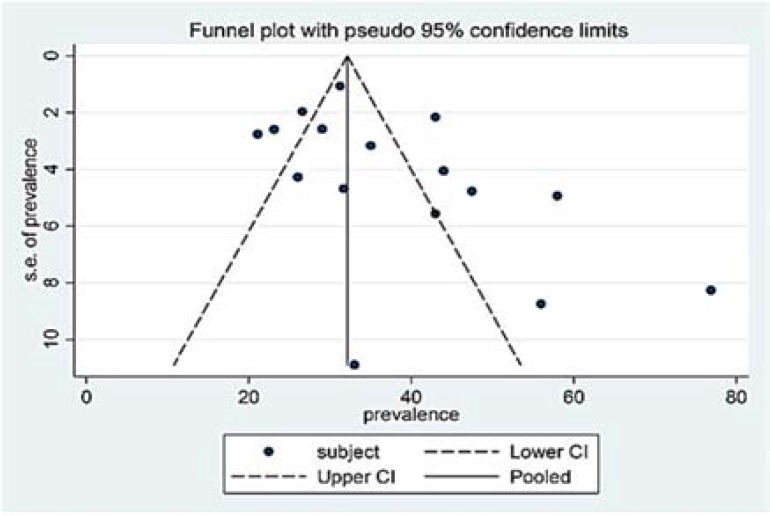
Funnel plot for the included studies with regards to the “subjective” group

**Figure 7:**
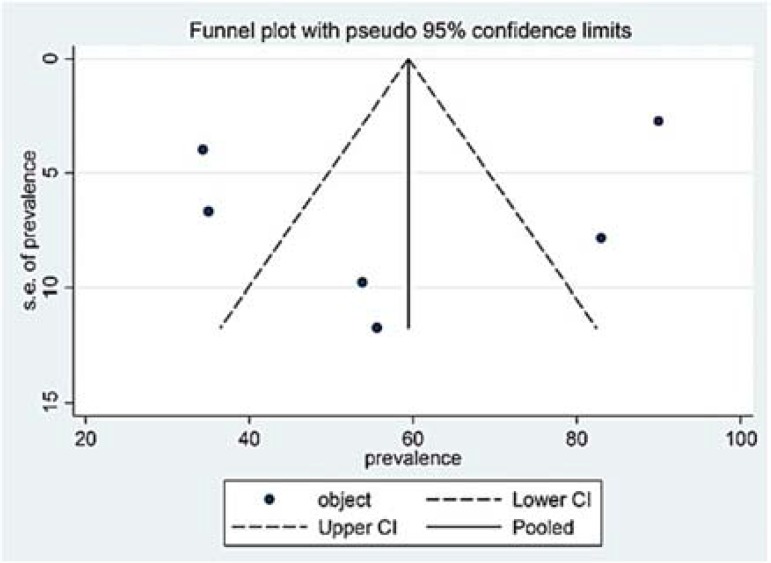
Funnel plot for the included studies with regards to the “objective” group

***Publications bias*** *: *We used Funnel plot to indicate the bias of the publications. The graphs demonstrated that the data were not symmetrical for the subjective and objective groups (Figures 6 and 7); hence, there was a bias in the publications of the studies indicating lack of published articles and the inaccessibility of authors to certain papers or the results of the required studies.

## Discussion

The current study was conducted with the aim of performing a systematic review and meta-analysis regarding the general prevalence and effective parameters of dysphagia disorder in patients affected by MS. 

A total of 5495 patients with MS were considered for the dysphagia disorder in 22 different studies. The total prevalence rate of dysphagia in people affected by MS was 43.33%; in objective and subjective methods, however, this rate was higher, 58.47% and 37.21%, respectively. The highest prevalence rates (76.90% and 90.00%) belonged to groups evaluated by the subjective and objective methods, respectively. Because more studies were added in the current study, this rate was contradictory to the similar previous research.^5^ The diversity associated with the reported rate of prevalence can be due to different diagnostic methods for the disease, sample size, year of publication of the study, regional differences, duration of the disease, or its acute conditions.

Various methods have been used to diagnose dysphagia in MS-affected patients for the subjective group. The tool that has had the most applications in finding the rate of prevalence is called “DYMUS” (DYsphagia in MUltiple Sclerosis), which is a questionnaire with 10 questions for screening dysphagia, that is filled by patients.^14^ 6 out of the 16 subjective studies and 4 among the 6 final studies done in 2013 employed DYMUS. Based on DYMUS, the average prevalence of dysphagia has somewhat increased since 2013. The results conduce to the growth of studies regarding dysphagia epidemiology and promote knowledge to deglutition situations in patients affected by MS.

**Figure 8:**
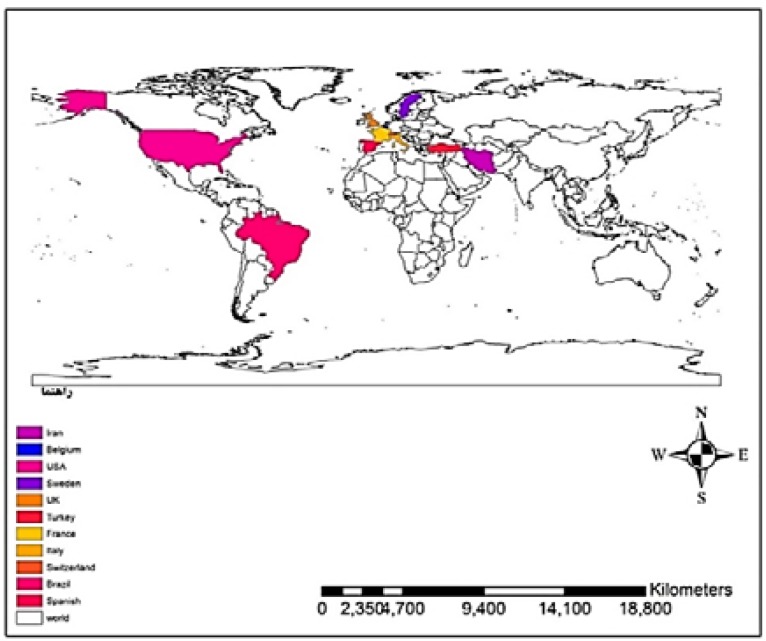
Geographical dispersion of the studies about dysphagia prevalence in multiple sclerosis (MS)-affected patients

As previously stated in the “Results” section, compared with subjective methods, the rate of dysphagia prevalence is higher in the studies conducted by objective method. According to the inclusion criteria, we were able to include and analyze 6 studies by the objective method. Videofluoroscopic swallowing study (VFSS), fiberoptic endoscopic evaluation of swallowing (FEES), and electromyography (EMG) techniques were employed in 3, 2, and 1 studies, respectively, among the 6 studies. VFSS is, therefore, the most used and reliable method for the diagnosis. The highest percentage of prevalence was obtained by the objective method (90%), a gold approach to identifying aspiration and dysphagia disorders. However, the fact is that the sample size in studies by the objective method was less than that in the subjective method; thus, the obtained results cannot be discussed with proper certainty as far as this factor is concerned. A sudden (insignificant) increase in dysphagia disorder was observed in the results of meta-regression, due to the considered duration, particularly since 2013, and the use of DYMUS which is not of accurate diagnosing sensitivity as it uses the reports by patients, and also screening nature of this checklist, such that it showed rate of dysphagia in the affected patients in a study to be 3.5 times the real value.^7^ Meta-regression analysis showed that dysphagia prevalence in studies with a sample size of over and less than 500 people was 33.6% and 45.1%, respectively, hence showing the importance of sample size. There were only 3 studies, out of the 22 considered studies, with a sample size of over 500 people, which may lead to diversity in the required results. The inhomogeneity in the studies and the different quality of the studies are the possible reasons for the reduction in the rate of prevalence. The geographical dispersion of the considered studies is yet another possible reason, since out of the 22 articles, 13 are from different European countries, 4 are from Iran, 2 are from the USA, and the remaining 3 are from Brazil (Figure 8). The highest and lowest rates of dysphagia prevalence are respectively reported from Brazil and Iran. In line with the previous studies,^5^^,^^12^^,^^27^ a positive correlation was observed between the prevalence of dysphagia with the severity of the disease and the MS stages. In contrast to a certain previous research^5^, on the other hand, a negative correlation was found between the prevalence of dysphagia and the duration of the disease, which may be due to the use of different diagnostic methods in studies, and also considering that in literatures, there were limited reports of the characteristics of patients with MS, we should be careful while interpreting the results. Thus, extensive studies are required in future to investigate the prevalence of dysphagia with similar credible tools, considering larger sample sizes, with simultaneous multi-central studies in various countries of different continents.

Regarding our systematic review of the studies, we encountered various limitations. It should primarily be noted that no unique diagnosing technique exists for dysphagia in either objective or subjective methods. Thus, it is recommended that one accurately consider, evaluate, and compare the diagnosing methods in future studies in order to get the best tools for diagnosing dysphagia. Also, our systematic analyses were mainly done according to cross-sectional studies, and lack of cohort and case-control studies can have adverse effects in causal conclusions. Hence, it is essential that such studies be designed and executed in future studies. The final point is that the considered population was limited to certain developed European countries and three other countries. Therefore, due to non-pervasive studies all over the world, especially in Africa and Oceania, the obtained results cannot be discussed reliably and represent the whole world in this regard. It is, therefore, required that we design and coordinate the studies in future to deal with the prevalence of dysphasia on a global scale.

## Conclusion

This study obtained the prevalence rate of dysphagia in patients affected by MS all over the world. It also considered the prevalence of the disease by different diagnosing methods, where it was found that dysphagia prevalence by taking objective method into account is more than that by the subjective method. Thus, it seems necessary to prioritize diagnosing analyses by the objective technique. However, more extensive studies with higher rates of evidence are essential for a better understanding of global epidemiology regarding dysphagia.

## Notes:

**How to cite this article:** Aghaz A, Alidad A, Hemmati E, Jadidi H, Ghelichi L. Prevalence of dysphagia in multiple sclerosis and its related factors: Systematic review and meta-analysis. Iran J Neurol 2018; 17(4): 180-8.
